# Terminal 6q27 Microdeletion Syndrome: A Case Report

**DOI:** 10.7759/cureus.31037

**Published:** 2022-11-02

**Authors:** Maycoll Ferreira Vieira, Daniela Carvalho, Filipa Valentim, Diogo M Branco, Helena Martinha Marinho

**Affiliations:** 1 Family Medicine, Madalena’s Health Center, Pico Island Health Unit, Madalena, PRT; 2 Family Medicine, Angra do Heroísmo’s Health Center, Terceira Island Health Unit, Angra do Heroísmo, PRT; 3 Family Medicine, Horta’s Health Center, Faial Island Health Unit, Horta, PRT; 4 Family Medicine, Praia da Vitória’s Health Center, Terceira Island Health Unit, Praia da Vitória, PRT

**Keywords:** terminal chromosome 6q27 deletion, cerebral ventricular asymmetry, child, scoliosis, developmental delay, facial asymmetry, congenital, syndrome, chromosome deletion, chromosome 6

## Abstract

A 10-year-old female with a history of global developmental delay (reduced concentration, cognitive impairment, and difficulty in reading and writing), scoliosis, aggressiveness, toe walking, and brain malformations was observed in the pediatric development outpatient consultation of Hospital de Santo Espírito da Ilha Terceira (HSEIT), Azores, Portugal. A genetic study was carried out and showed a terminal 6q27 microdeletion, a rare disorder. Being so rare, it’s important to share with the wider medical community any of such cases so early diagnosis can occur and interventions may be developed.

## Introduction

Terminal 6q27 microdeletions are rare disorders involving a deletion at the long arm of chromosome 6 [1], specifically at the 6q27 terminus. The 6q27 region encodes genes important for brain development [2], and terminal deletions of these genes are associated with intellectual disability, epilepsy, hypotonia, cardiac defects, retinal and ear abnormalities, facial dysmorphisms, and malformations of the brain (such as agenesis of the corpus callosum, periventricular heterotopia, polymicrogyria, cerebellar malformations, and hydrocephalus), spinal cord, and vertebrae [3].

Being a rare disorder, literature is scarce on the topic, being still a poorly understood subject. We report a case of a 10-year-old female diagnosed with a terminal 6q27 microdeletion syndrome, observed in 2021 in the Hospital de Santo Espírito da Ilha Terceira (HSEIT), Azores, Portugal.

## Case presentation

A 10-year-old female had been followed in the pediatric development outpatient consultation of HSEIT since 2018. She was initially referred from the general pediatrics outpatient consultation for global developmental delay and suspicion of Williams syndrome. She was a full-term baby born out of nonconsanguineous parents, being the first child and first pregnancy of the mother and having three paternal half-siblings. Her parents were separated, her father living out of the country at the time of observation. No known family history of developmental disorders existed.

She was born at 37 weeks, with a normal weight and Apgar score. A combined first-trimester prenatal screening (comprising an ultrasonography measuring the crown-rump length, the nuchal translucency, and nasal bone assessment; serum screening of free beta-human chorionic gonadotropin {bhCG}; and pregnancy-associated plasma protein A {PAPP-A}) was performed, having returned a negative result (low risk for Down, Edwards, and Patau syndromes). No cell-free fetal DNA (cffDNA) testing or amniocentesis was performed. After birth, a metabolic disease screening was performed, ruling out pathologies such as congenital hypothyroidism, cystic fibrosis, amino acid disorders, organic acidurias, and inherited fatty acid beta-oxidation disorders.

She had a normal stature and weight since then (3rd-15th percentile). She started walking and talking at two years old, never needing speech therapy. She showed difficulties in concentration and comprehension since kindergarten and some aggressiveness since she was three years old. At school, she underperformed with the normal curriculum, having started an adapted one in the second grade. She was followed by pedopsychiatry and psychology and medicated with risperidone 1 mg and methylphenidate 10 + 5 mg, at the time of observation.

After ruling out metabolic disorders, a genetic study (array comparative genomic hybridization {aCGH}) took place finding a terminal deletion in the long arm of chromosome 6, in 6q27, of around 530 kb, involving 10 genes. According to the geneticist, there are two patients with similar deletions on the DatabasE of genomiC varIation and Phenotype in Humans using Ensembl Resources (DECIPHER) database and some in literature, with similar deletions and showing intellectual disabilities, hypotonia, global developmental delay, brain abnormalities, and epilepsy. It was not considered to be inherited from her parents, even though gonadal mosaicism couldn’t be excluded. The probability of transmission from the patient’s descendants was determined to be 50%.

During the consultation, she was observed walking around the room and talking and playing by herself, as well as reproducing dialogues she knew from television and movies. She changed rapidly between activities, not being able to concentrate on one task for long, apparently performing best in environments with less stimuli. She answered when called and talked normally.

**Figure 1:**
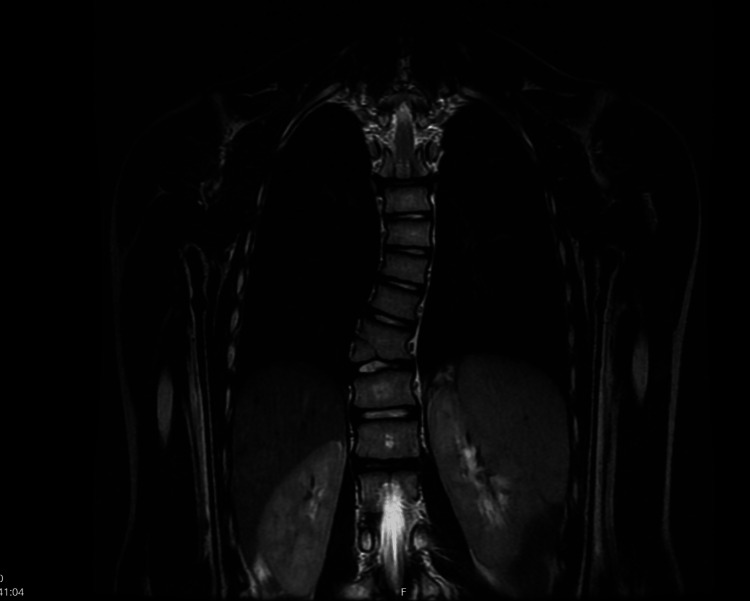
Magnetic resonance image showing congenital scoliosis.

She showed extreme difficulty in reading, being able to read only some words and not recognizing every letter, and knew only how to write her first name. She also presented toe walking. Physically, she did not show any abnormality, except for scoliosis curving to the right, and afterward was observed to have a Cobb angle of 18° (Figure 1) and facial asymmetry.

**Figure 2:**
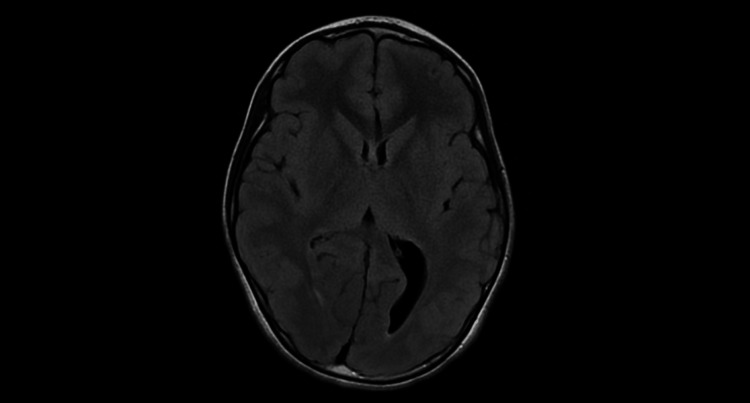
Brain MRI showing asymmetric ventriculi. MRI: magnetic resonance imaging

An echocardiogram was performed, with no abnormalities detected. A cranioencephalic magnetic resonance imaging (MRI) showed an enlarged corpus callosum and asymmetric ventriculi (Figure 2). Subependymal heterotopias were observed on the temporal end of the lateral right ventricle. Possible polymicrogyria was observed around the left temporal area, with peculiar (sic) sulci.

## Discussion

The case presented describes a patient with a terminal deletion in chromosome 6, specifically in region 2 and band 7. Terminal deletions of chromosome 6 are rare findings and can resemble other genetic syndromes (in our case, it was initially hypothesized, through physical examination alone, by her pediatrician to be a case of Williams syndrome), since they can present with a plethora of different phenotypes [2], none, to our current knowledge, pathognomonic to them.

Typical characteristics found in literature include, as previously mentioned, intellectual disability, epilepsy, hypotonia, cardiac defects, retinal and ear abnormalities, facial dysmorphisms, agenesis of the corpus callosum, periventricular heterotopia, polymicrogyria, cerebellar malformations, hydrocephalus, and spinal cord and vertebra malformations.

Our patient showed some, but not all, of those characteristics, most notably intellectual disability, facial asymmetry, scoliosis, asymmetric brain ventriculi, polymicrogyria, and subependymal heterotopias. The fact that she showed toe walking might also hint at a level of cerebellar malformation. We speculate that possibly some frontal involvement might be present, considering her sudden bursts of aggression (Table 1).

**Table 1:** Typical versus patient characteristics.

Typical characteristics found in literature	Patient characteristics
Intellectual disability	✓
Facial dysmorphism	✓
Periventricular heterotopia	✓
Polymicrogyria	✓
Spinal cord and vertebra malformations	✓
Cerebellar malformations	Likely
Epilepsy	
Cardiac defects	
Hypotonia	
Retinal abnormalities	
Ear abnormalities	
Agenesis of the corpus callosum	
Hydrocephalus	
-	Subependymal heterotopias
-	Aggression (possible frontal involvement)

There’s no specific treatment protocol for this syndrome, with symptomatic care being the basis of management. Regarding our patient, school support, psychology, pedopsychiatry, and physiatry were important pillars of treatment, as well as continued monitoring in the pediatric development outpatient consultation. Vigilance and genetic testing are essential, as well as a prompt symptomatic intervention.

## Conclusions

Terminal 6q27 deletions are rare but have important phenotypic consequences for the patients and can cause marked distress to families. An early diagnosis, vigilance, and management are crucial for the patient’s well-being. More research is needed, considering the scarce literature available, which is nonetheless justified because of the rarity of the condition.
